# *Tamarix articulata* Induced Prevention of Hepatotoxicity Effects of In Vivo Carbon Tetrachloride by Modulating Pro-Inflammatory Serum and Antioxidant Enzymes to Reverse the Liver Fibrosis

**DOI:** 10.3390/antiox11091824

**Published:** 2022-09-15

**Authors:** Abdullah M. Alnuqaydan, Abdulmajeed G. Almutary, Mohammed A. Alsahli, Sulaiman Alnasser, Bilal Rah

**Affiliations:** 1 Department of Medical Biotechnology, College of Applied Medical Sciences, Qassim University, Buraydah 51452, Saudi Arabia; 2 Department of Medical Laboratories, College of Applied Medical Sciences, Qassim University, Buraydah 51452, Saudi Arabia; 3 Department of Pharmacology and Toxicology, Unaizah College of Pharmacy, Qassim University, Buraydah 51452, Saudi Arabia; 4 Iron Biology Group, Sharjah Institute of Medical Research, University of Sharjah, Sharjah 27272, United Arab Emirates

**Keywords:** antioxidant, fibrosis, hepatotoxicity, inflammation, *Tamarix articulata*

## Abstract

This study evaluates the hepatoprotective activity of a *Tamarix articulata* extract against carbon tetrachloride-mediated hepatotoxicity in Wistar rats. Our results demonstrated that the oral administration of *Tamarix articulata* extract (50 mg/kg b.w.) significantly restored the serum levels of liver enzymes and antioxidant parameters (superoxide dismutase, catalase, glutathione reductase, and thiobarbituric reactive substances). Histopathology analysis revealed that *Tamarix articulata* extract significantly reduced hepatic fibrosis by inhibiting the necrosis of hepatocytes. Furthermore, serum pro-inflammatory (tumor necrosis factor-alpha, tumor growth factor-beta, and interleukin-6) markers were significantly restored. However, the anti-inflammatory cytokine adiponectin levels increased to normal levels in the group treated with *Tamarix articulata* extract. Additionally, we observed diminished reactive oxygen species production and the depolarization of mitochondrial membrane potential in hepatocytes extracted from animal livers treated with *Tamarix articulata* extract. Our findings suggest that *Tamarix articulata* extract prevents liver fibrosis induced by carbon tetrachloride and decreases the necrotic population of hepatocytes. These events restored the antioxidant enzymatic activity, serum levels of liver enzymes, and pro-inflammatory markers to their normal levels.

## 1. Introduction

The liver performs a key role in the regulation of numerous biochemical functions associated with metabolism [1]. Liver injury is a multi-factorial disease caused by environmental and chemical toxins, drugs, and alcohol intake, which induce oxidative stress, leading to complicated pathophysiology. Typically, the end result of this is cirrhosis and hepatocellular carcinoma (HCC) [2,3]. The liver is rich in mitochondria that aid in aerobic metabolism through the electron transport chain (ETC). During the process of oxidative phosphorylation, reactive oxygen species (ROS) are produced, and this means that hepatocytes are highly susceptible to oxidative stress, which ultimately leads to hepatocellular damage. Owing to a number of side effects of the existing therapeutic modalities used for liver diseases, the management of hepatocellular diseases is often poor. Thus, the need to develop efficient, effective, and reliable hepatoprotective drugs from plant sources with fewer adverse effects is seemingly important [4] This notion is supported by various reports that suggest that vegetarian diets and fruits from plant sources are rich in antioxidants. The consumption of such a diet drastically curtails the risk of developing chronic hepatocellular disorders [5].

Environmental toxicants have been documented as inducing oxidative stress by promoting ROS production, which leads to liver injury, necrosis, and tissue damage. [6]. One of the potent environmental toxicants is carbon tetrachloride (CCl_4_). Upon the administration of CCl_4_into the body orally or through systemic circulation, the detoxifying enzyme cytochrome P-450 present in the liver converts CCl_4_into a more toxic form called the trichloromethyl radical (CCl_3_^+^) through a process called biotransformation [7]. In the presence of oxygen, CCl_3_^+^ is converted into a highly reactive radical form called trichloromethylperoxy (CCl_3_OO^+^) [8]. These highly reactive free radical species covalently bind with phospholipid membranes, which in turn induce lipid peroxidation that firstly impairs cell membrane integrity and secondly enhances the permeability of cells, thus causing severe damage to them [9]. Owing to its toxic effects, the use of CCl_4_is restricted, yet it is still one of the most widely used chemicals for the induction of hepatotoxicity in animal studies. It serves to evaluate the modulation of the pro-inflammatory and anti-scavenging effects of plant products abundant in antioxidants that could act as promising hepatoprotective agents [10].

Recently, antioxidants obtained from various plant remedies have been introduced in therapeutics for liver fibrosis [11]. Such anti-fibrotic therapies are used to modulate oxidative stress and have been entered into clinical trials [12]. Morin, a flavonoid compound with antioxidant activity, primarily isolated from *Maclura pomifera*, prevents CCl_4_-mediated liver fibrosis by attenuating the levels of nitric oxide (NO) and MDA to restore the GSR of hepatocytes to its normal levels [13]. Silymarin—a mixture of flavonolignan compounds—exhibits promising antioxidant activity. When administered orally, a 100 mg/kg silymarin dose prevents CCl_4_-mediated liver fibrosis by reducing the MDA levels, restoring the GSR activity of hepatocytes, and preventing the depolarization of MMP to counter oxidative stress [14]. Epigallocatechin (EGCG) is another polyphenolic compound with antioxidant activity that prevents liver fibrosis in a plethora of CCl_4_-mediated animal models [15]. Together, these studies suggest that natural antioxidant compounds show much potential to reverse fibrosis by attenuating oxidative stress, and preclinical investigations as part of clinical trials are required for future therapeutics.

*Tamarix articulata* (TA) is a halophytic plant (belongingto the family *Tamaricaceae*) commonly found in the deserts of Saudi Arabia. Traditionally called “Athal” in the Arabic language, TA grows extremely well in drought, harsh, and arid conditions. The plant grows to a height of 15 m and a girth of 2 m, as described in our previously published study [16]. Ethnobotanical studies have revealed that TA has been extensively used as folk medicine by the Tafilalet, a tribal population in the South-Eastern area of Morocco. Traditionally, the plant is used to treat various ailments, such as gastrointestinal, skin, heart diseases, and other ailments [17]. Phytochemical analysis revealed the major constituents of TA extract thatdisplay pharmacological activities, as mentioned in our previous study [18]. Crude extracts of the plant have been reported to exhibit anticancer activities by inhibiting cell viability in various types of cancer cells [19]. Owing to the large amount of polyphenolic and flavonoid compounds, the methanolic extract of TA is apromising antioxidant with antiproliferative activities, as stated in our previous research [16,20]. Although TA exhibits some promising pharmacological activities, including antioxidant, antiproliferative, and antilipidemic activities [16,18,19,20,21], there is not a single report suggesting that TA extract exhibits hepatoprotective activity. Therefore, the current study evaluates its hepatoprotective activity against the well-established carbontetrachloride-induced hepatotoxicity in Wistar rats. We found that TA extract reverses liver fibrosis induced by CCl_4_in animals and restores pro-inflammatory as well as serum enzymes and antioxidant enzymes. Together, these results suggest that TA has a promising hepatoprotective effect and has much potential as a remedy against fibrosis and liver-associated diseases. 

## 2. Materials and Methods

### 2.1. Chemicals, Reagents, and Kits 

Carbon tetrachloride (CCl_4_) (#PHR1063), silymarin (#S0292), and other chemicals and reagents were purchased from Sigma Aldrich Chemicals Co., St. Louis, Missouri, United States. Kits for the pro-inflammatory (TGF-β #ab119558, TNF-α #ab236712, IL-6 #ab234570, and adiponectin #ab239421) and anti-inflammatory cytokine markers (SOD #ab65354, CAT #ab118184, GSR #ab65322, and TBARS/MDA #ab238537), liver function test (LFT) (AST #ab263882, ALT #234579, ALP #ab83369, and Bil #ab235627) enzymes to detect free radical scavenging activity, mitochondrial membrane potential (MMP) (MMP #ab113852), and ROS (ab #ab186027) detection were purchased from Abcam, Cambridge, United Kingdom.

### 2.2. Plant Material

TA plant was collected in August 2019 from Qassim Province in Saudi Arabia. The leaves of the TA plant were air-dried in the shade to remove all of the moisture [20]. The data from our previous work identified the plant extracts, while the phytochemical analysis of major constituents of TA extracts displayed various pharmacological activities. A comparative assessment of different parts of the plant was conducted as described in our recently published studies [18,20,21]. 

### 2.3. Preparation of Extract

Methanolic extract of TA was prepared as per the standard protocol [21]. Dry TA leaves were collected from the floor and washed with distilled water. After cleaning, the TA leaves were shade-dried for 10 days to ensure complete drying followed by grinding in a kitchen blender to produce a fine powder. The dried TA leaf (100 g) powder was soaked in methanol (300 mL). Using the manual method of extraction, the mixture of dry leafpowder in methanol was constantly stirred on amagnetic stirrer at room temperature for 5 days. The obtained extract was filtered through Whatman filter paper and was concentrated by evaporating the solvent to attain a fine residue powder. The yield of the methanolic extract of the dry leaves of TA amounted to 8.33% and the dry powder of the extract was stored at 4 °C for future use.

### 2.4. Liquid Chromatography–Mass Spectrometry (LC–MS) Metabolomic Analysis and Data Processing

The analysis was carried out as described in our recently published studies [18,20,21]. LC–MS metabolomic analysis was undertaken consisting of an ACQUITY UPLC I-Class System (Waters Technologies, Milford, MA, USA) coupled with a 6500Qtrap(AB Sciex, Concord, ON, Canada). Chromatographic separation was completed on a Zorbax XDB C18 column (2.1 × 150 mm. 3.5 µm) (Agilent, Santa Clara, CA, USA) maintained at 40 °C with a flow rate of 300 µL/min. The mobile phase consisted of A (0.1% formic acid in HPLC grade water) and B (0.1% formic acid in HPLC grade acetonitrile). The linear gradient elution was as follows: 2% B (from 0 to 2), 95% B (from 2 to 24), 95% B (held for 2 min), and then 4 min equilibration time. Electrospray ionization mass spectra (ESI-MS) were acquired in the positive mode (ES+), with an electrode voltage of 5500 V. The declustering potential was set to 90 V and the entrance potential was 10 V. Nitrogen was used as the curtain gas (30 psi) and nebulizer gas on the MS. Spectra were collected with a mass range of 100–900 m/z. Data files from the LC were converted to MZxml format using MS Convert (ProteoWizard 3.0.20270). Analysis of the data was conducted using MZ mine software (version 2.53). After importing the data into the MZ mine, a minimum intensity cutoff of 1000 was applied and the retention time was adjusted with a tolerance of 0.2 min. Adjusted peaks were then aligned to one mass list to facilitate identification and comparison. The KEGG Database was used to identify compounds of interest in the finalized list based on m/z with a tolerance of 30 ppm.

### 2.5. Experimental Animals

Male Wistar rats weighing 100–110 g were procured from KAUST, Saudi Arabia. All animals (Wistar rats) were acclimatized in the institute animal house for at least one week prior to experimentation with a 12 h dark and light cycle at room temperature. Animals were fed with a standard pellet diet and water ad libitum. This research was endorsed by the Ethics Committee of the College of Applied Medical Sciences, Qassim University (cams1-2019-1-14-s-3360).

### 2.6. CCl_4_-Induced Hepatotoxicity

The Wistar male rats acclimatized in the animal house were randomly grouped into 7 groups, with each group having 6 rats (42 Wistar rats) per experiment (n = 3, meaning 126 animals for the whole study). Animals were orally dosed with CCl_4_as mentioned previously [22,23], and other respective concentrations of TA extracts (30, 40, and 50 mg/kg b.w.) using gastric gavage without the administration of any anesthesia agent. Group A was designated as normal, without any chemical or extract being administered. Group B was designated as the CCl_4_-treated group and the animals were administered 40% CCl_4_mixed in olive oil orally for 3 alternate days a week for 8 weeks. Group C was designated as 40% CCl_4_mixed in olive oil and TA extract 30 mg/kg administered orally for 3 alternate days a week for 8 weeks. Group D was designated as 40% CCl_4_mixed in olive oil and TA extract 40 mg/kg administered orally for 3 alternate days a week for 8 weeks. Group E was designed as 40% CCl_4_mixed in olive oil and TA extract 50 mg/kg administered orally for 3 alternate days a week for 8 weeks. Group F was designated as 40% CCl_4_mixed in olive oil and TA extract 60 mg/kg administered orally for 3 alternate days a week for 8 weeks. Group G was designed as 40% CCl_4_mixed in olive oil and 100 mg/kg b.w. silymarin [24] administered orally for 3 alternate days a week for 8 weeks (Table 3).

To evaluate the effective dose of TA extract with the least toxicity to the animals, we intended to perform a preliminary experiment on animals. This was performed to optimize the dose of TA extract that could exhibit effective hepatoprotective activity with less deleterious effects on animals. After properly acclimatizing the animals, we categorized them randomly into seven groups, as shown in Table 3. The disease control (Group B) animals were orally administered 200 µL 40% CCl_4_dissolved in olive oil for three alternate days a week for 8 weeks, whereas the control group (Group A) animals were orally administered 200 µL of vehicle. The other groups (Group C, D, E, and F) were orally administered 200 µL of 40% CCl_4_dissolved in olive oil and 200 µL of TA extract with varying doses (30, 40, 50, and 60 mg/kg b.w., respectively) for three alternate days a week for 8 weeks. However, Group G animals were orally administered 200 µL of standard silymarin compound for three alternate days a week for 8 weeks; this was in addition to 200 µL of40% CCl_4_dissolved in olive oil. To evaluate the effective dose in terms of the hepatoprotective effect and safe toxicity in animals, we observed the hepatoprotective activity of TA extract against CCl_4_-mediated liver toxicity in a dose-dependent manner (Tables 4 and 5). Intriguingly, our results suggest that the ideal activity of TA extract was determined at 50 mg/kg b.w. (Group E), and the results were significantly compared with those of standard silymarin (Group G). When tested at 60 mg/kg b.w. (Group F), TA extract displayed toxicity-associated symptoms in animals, such as lethargy, diarrhea, muscle tremors, loss of appetite, etc., and they died before the completion of the study (Table 4 and Table 5). Collectively, the preliminary optimization results suggest that a 50 mg/kg b.w. oral dose of TA extract has the maximum hepatoprotective effect against CCl_4_-mediated liver toxicity.

### 2.7. Evaluation of Liver Function Test

Following the completion of the treatment schedules, the animals in each group were sacrificed following methods approved by the Ethics Committee of the College of Applied Medical Sciences, Qassim University (cams1-2019-1-14-s-3360). Once the study was completed, diethyl ether was used to anesthetize the animals, followed by cervical dislocation and operating procedures to extract internal organs for the current study. The blood (1.0 mL) was collected from the tail vein before sacrificing the animals. The collected blood was allowed to clot and was centrifuged at 3000 rpm and 4 °C for 10 min [25]. The supernatant (serum) was collected in sterile microfuge tubes and stored at −80 °C for the analysis of various biochemical parameters. The liver function enzyme levels of alkaline phosphatase (ALP), alanine transaminase (ALT), and aspartate aminotransferase (AST), and the bilirubin levels in serum were estimated as previously described [26]. However, the quantification of pro-inflammatory cytokine markers (TNF-α, IL-6, and TGF-β) and the anti-inflammatory cytokine marker adiponectin levels were investigated with the use of an enzyme-linked immunosorbent assay (ELISA) kit. 

### 2.8. Histopathological Analysis

The liver tissue was extracted from the animals of each group (n = 6) and thoroughly cleaned in PBS at pH 7.4. A small chunk of liver tissue was excised and fixed in 10% formalin to preserve the morphology of the cells from putrification. After processing the samples for paraffin blocks, the thin paraffin sections were cut with ultra-microtome. The sections were subjected to hematoxylin–eosin for inflammation and Masson’s trichome staining to analyze the histopathological changes in the collagen fibers of the hepatic tissue using a microscope [27]. 

### 2.9. Detection of In Vivo Antioxidant Enzymes

Briefly, a small chunk of liver tissue (approximately 10%) was chopped into smaller pieces, followed by homogenization (Fisherband 150 efficient homogenizer, #15-340-167, Waltham, MA, USA) in a 2 mL sterile tube by using 1 mL of PBS (pH 7.4). The mixture obtained was subjected to centrifugation at 10,000 rpm for 10 min at 4 °C. The supernatant obtained was used to evaluate the antioxidant enzymes and pro-inflammatory markers. The enzymatic activity of catalase (CAT), superoxide dismutase (SOD), glutathione reductase (GSR), and malondialdehyde (MDA) per milligram of the protein concentration of hepatic tissue homogenate was estimated utilizing a spectrophotometric method (Bradford method) [28]. 

### 2.10. Determination of Reactive Oxygen Species (ROS)

After extracting the livers from the animals in each group (n = 6), part of the liver was excised, washed thoroughly with PBS followed by resuspension in culture media, and subjected to tissue dissociation with a tissue dissociator. After counting the hepatocytes using a hemocytometer (Neubauer chamber; #02-671-6, Waltham, MA, USA), 1 × 10^6^ hepatocytes obtained were exposed to DCFH-DA for 15 min at 37 °C in a 5% CO_2_incubator in the dark. The presence of free radicals or ROS converting DCFH-DA into dichlorofluorescein emits a green color; here, it was recorded with a fluorescence microplate reader at an excitation wavelength of 488 nm and emission wavelength of 525 nm [29]. 

### 2.11. Determination of Depolarization Mitochondrial Membrane Potential (MMP)

Briefly, 1 × 10^6^ hepatocytes obtained from the tissue dissociation process were exposed to JC-1 dye for 15 min at 37 °C in a 5% CO_2_incubator in the dark. Immediately, suspended stained cell mixtures were analyzed by flow cytometry to evaluate the depolarization of MMP with anexcitation wavelength of 488 nm and emission wavelength of 590 nm [29]. 

### 2.12. Statistical Analysis

All the results obtained represent the mean ± standard error of the mean (SEM), calculated and processed by one-way ANOVA. A *p*-value equal to or less than 0.05 was designated as significant.

## 3. Results

### 3.1. Phytochemical Analysis and Dose Optimization of TA Extract in Rats to Evaluate the Hepatoprotective Effect

**Figure 1:**
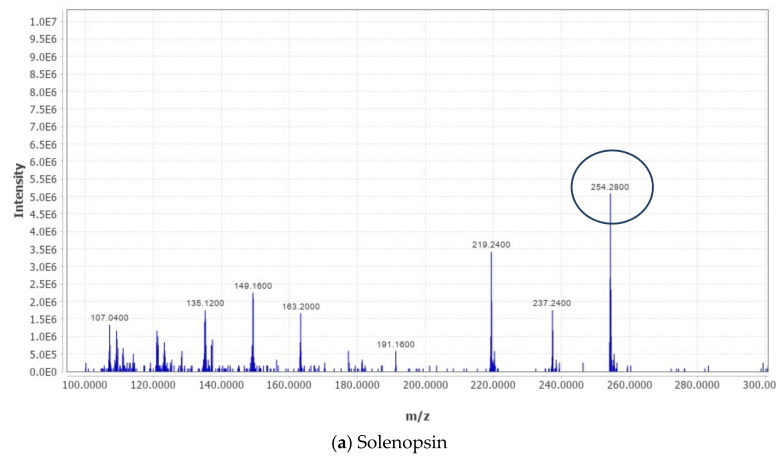

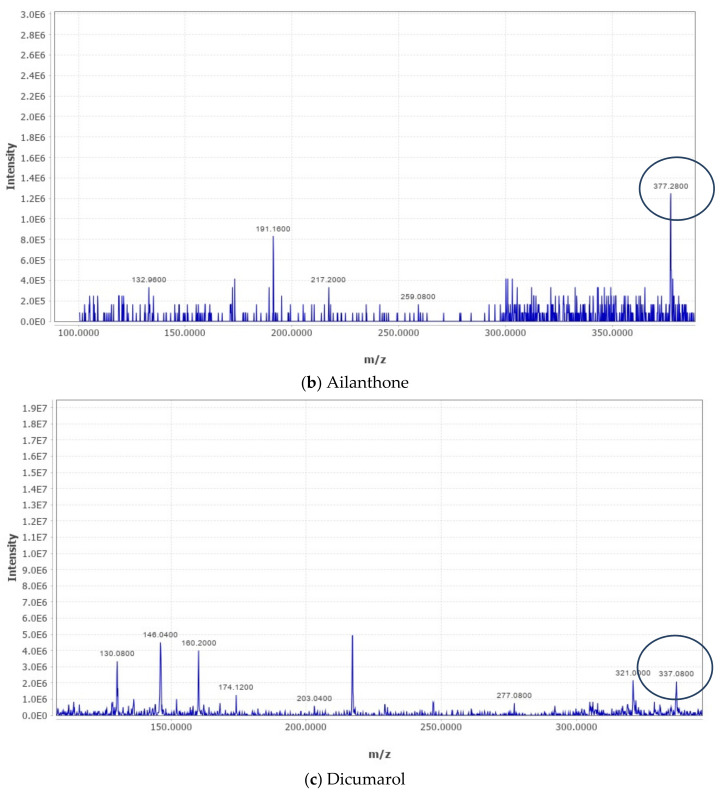

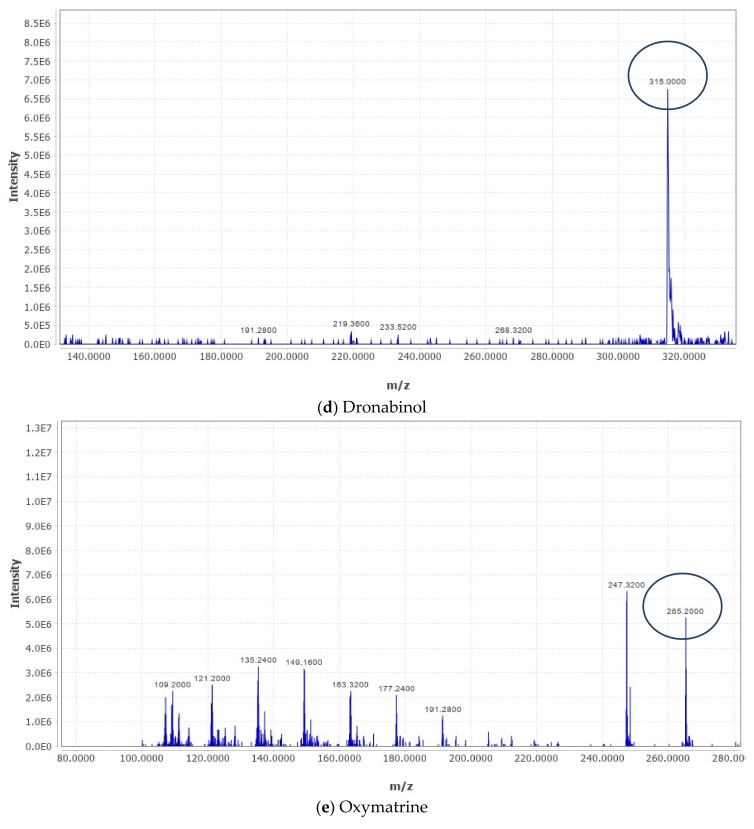

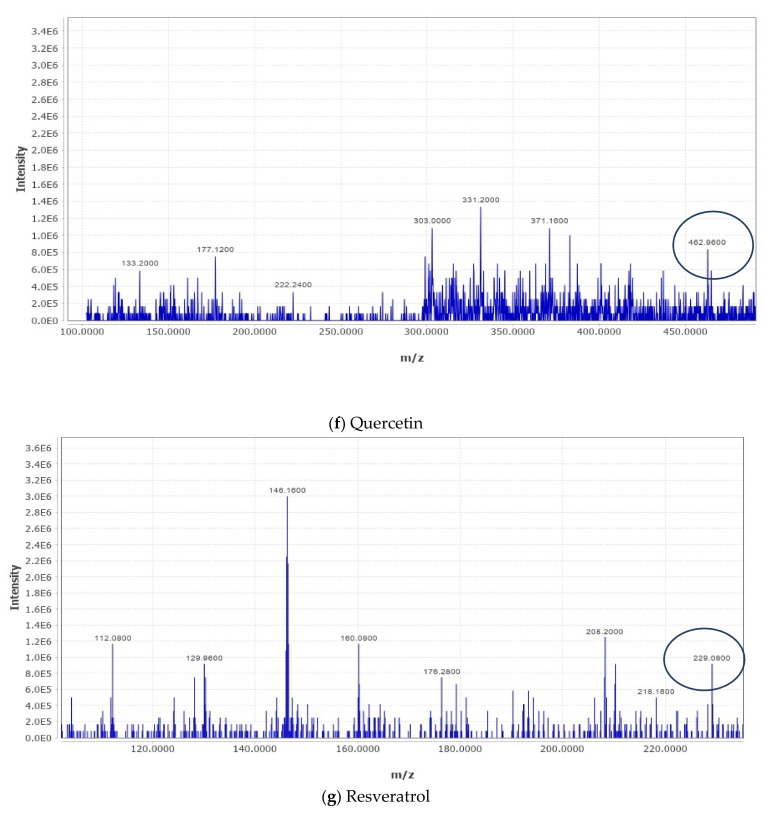

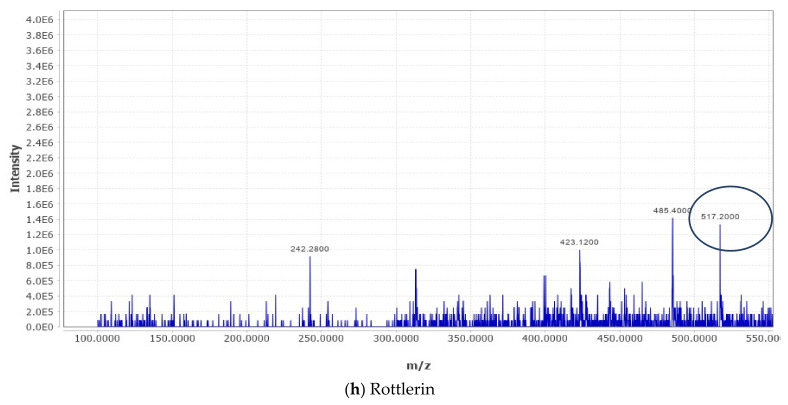
Chromatogram of key phytochemicals present in TA extract (this figure shows data from our recently published work [18,20,21]).

The phytochemical analysis of the methanolic extract of TA by LC-MS analysis revealedthat more than 200 compounds were identified (Table 1). The major compounds are summarized in Table 2 and their respective chromatograms are presented in Figure 1. The key phytochemicals identified from the methanolic extract of TA by LC-MS display anticancer activities against various cellular models and are summarized in Table 2.

We have performed the dose optimization and treatment schedule of different groups of animals in Table 3.

We have performed the analysis of antioxidant enzymes of liver tissues of animals treated with different concentrations of TA extract in Table 4.

We have performed the biochemical parameters of liver function test in Table 5.

### 3.2. Effect of TA Extract on the Biochemistry of The Liver

Serum biochemistry analysis revealed that a substantial amount of hepatocellular damage was induced after the oral administration of 40% CCl_4_mixed in olive oil, which was evidenced by a significant (*p* < 0.01) elevation in the levels of liver function enzymes (ALT, ALP, and AST) and bilirubin (Table 5) when compared with the control group. However, for animals in the group dosed with 50 mg/kg b.w. TA extract, significant (*p* < 0.01) recovery was observed in the liver enzymes and bilirubin level when compared with CCl_4_mixed in olive oil and 100 mg/kg b.w. standard silymarin for 8 weeks. Collectively, these results suggest that a significant recovery of the serum liver and antioxidant enzymes to normal levels was observed in animals treated with a 50 mg/kg b.w oral dose after the induction of hepatotoxicity with CCl_4_.

**Figure 2:**
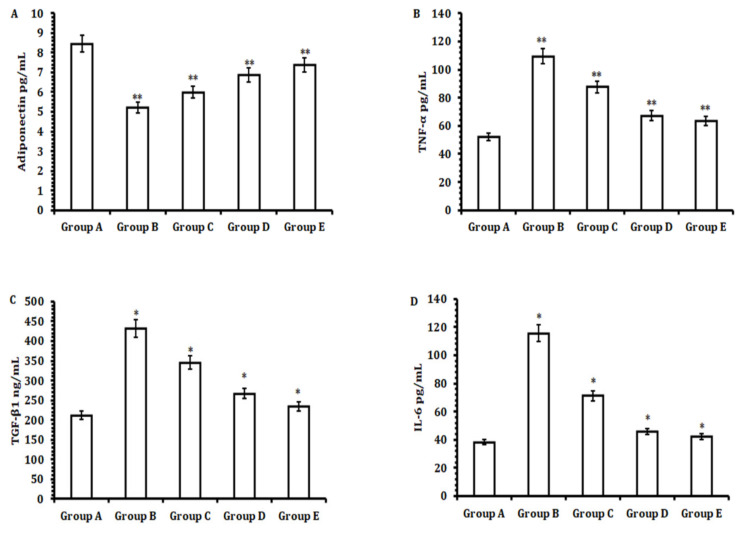
Effect of TA extract on anti-inflammatory (adiponectin) and pro-inflammatory cytokine markers (TNF-α, TGF-β, and IL-6) of liver tissues initially treated with CCl_4_to induce liver toxicity. (**A**) Adiponectin level, (**B**) TNF-α level, (**C**) TGF-β level, and (**D**) IL-6 level. The experiments were performed three times or more than three times, and the represented data are the mean values ± SEM. A *p*-value less than or equal to 0.05 was considered to be statistically significant; * *p* ≤ 0.05, ** *p* ≤ 0.01.

Next, we aimed to evaluate the pro-inflammatory markers in the serum samples of different animal groups with the objective of analyzing the hepatocellular inflammation induced by CCl_4_and recovery after treatment with TA extract. We observed a sharp decline in the serum concentration of adiponectin, an anti-inflammatory marker cytokine protein derived from adipocytes, and an increase in the serum levels of pro-inflammatory cytokine markers TGF-β, IL-6, and TNF-α when the animals were administered CCl_4_compared with the control group animals (Figure 2). However, after treating the animals with 50 mg/kg b.w. of TA extract, we observed a significant increase in the serum level of anti-inflammatory cytokine adiponectin (*p* < 0.01) (Figure 2A). Furthermore, analysis revealed that a significant decrease in the serum levels of pro-inflammatory cytokine (TNF-α, *p* < 0.01; IL-6, *p* < 0.05; and TGF-β, *p* < 0.05) (Figure 2B–D) was observed when animals were dosed with 50 mg/kg b.w. of TA extract when compared with the CCl_4-_treated group and control group. Intriguingly, we observed that the results with a 50 mg/kg b.w. oral dose of TA were as good as those with a 100 mg/kg b.w. oral dose of standard silymarin. Together, these results suggest that TA extract firstly restored the level of anti-inflammatory adiponectin and secondly decreased the levels of the pro-inflammatory cytokine markers TNF-α, IL-6, and TGF-β. These were as good as those obtained for silymarin, a standard compound able to manage CCl_4_-mediated hepatocellular inflammation.

### 3.3. Effect of TA Extract on Histopathology of Hepatic Tissue

**Figure 3:**
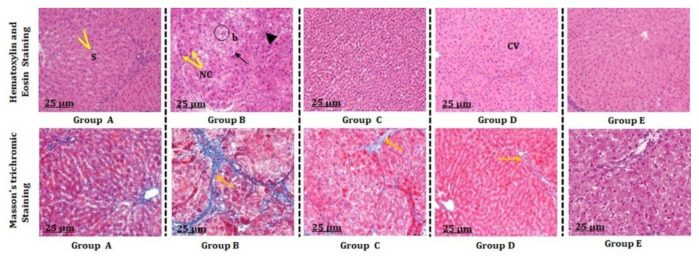
Effect of TA extract on the microscopic analysis of hepatic tissue sections of animals initially treated with CCl_4_. Hematoxylin and eosin stain of hepatic tissues in control (Group A), CCl_4_-treated animals (Group B), CCl_4_+30 mg/kg b.w. TA extract-treated animals (Group C), CCl_4_+ 40 mg/kg b.w. TA extract-treated animals (Group D), and CCl_4_+ 50 mg/kg b.w. TA extract-treated animals (Group E). Masson’s trichrome staining in control (Group A), CCl_4_-treated animals (Group B), CCl_4_+ 30 mg/kg b.w. TA extract-treated animals (Group C), 40 mg/kg b.w. TA extract-treated animals (Group D), and CCl_4_+ 50 mg/kg b.w. TA extract-treated animals (Group E). Bar scale = 25 μm. Magnification × 100.

Histopathological analysis revealed that remarkable hepatocellular damage occurred in animals administered with CCl_4_ (Group B) when compared with the untreated group (A). Further microscopic analysis demonstrated that a significant necrotic population of hepatocytes with a deformed shape, large number of vacuolizations, infiltration of inflammatory cells, portal biliary damage, and hepatocyte blooming was observed in Group B animals when compared with control Group A. In the latter, hepatocytes with a definite polyhedral shape and a prominent nucleus and other cellular organelles were observed. However, in Group E, where animals were orally dosed with 40% CCl_4_mixed in olive oil or 50 mg/kg b.w. TA extract, they showed significant protection against CCl_4_-mediated hepatotoxicity with diminished necrosis of hepatocytes, infiltrated cells with less vacuolization and hepatocyte blooming, and hepatocytes were able to maintain their proper shape and architecture (Figure 3, Table 6). Further, Masson trichome staining revealed that the liver section of the CCl_4_-treated group showed significant fibrosis, which was characterized by the enlargement of collagenous tissue in and around the portal tract (Table 6). Interestingly, liver sections of the animals treated with 50 mg/kg b.w. TA extract (Group E) exhibited considerably little fibrotic tissue and low deposition of collagen in and around the portal tract compared with the groups treated with lower doses of TA extract (30 and 40 mg/kg b.w.; Groups C and D, respectively) (Figure 3). Collectively, these results suggest that TA extract demonstrates promising hepatoprotective activity against CCl_4_-mediated hepatotoxicity.

### 3.4. TA Extract Restores Antioxidant Enzymes against CCl_4_-Mediated Oxidative Stress and Prevents Lipid Peroxidation

**Figure 4:**
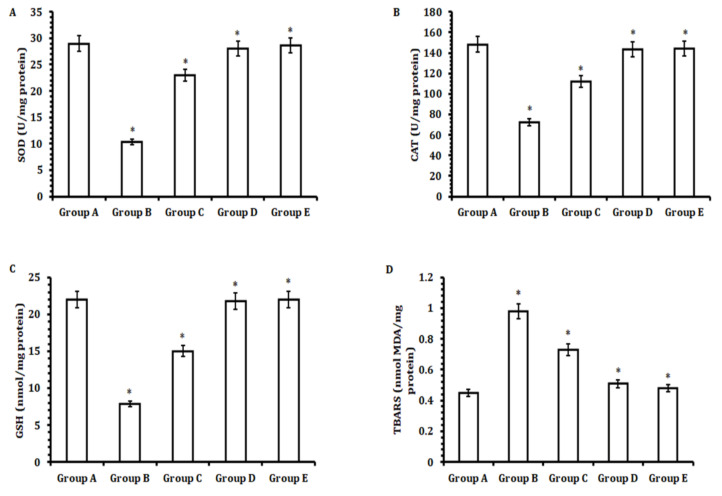
Effect of TA extract on the antioxidant enzymes (SOD, CAT, GSR, and MDA) of liver tissues initially treated with CCl_4_to induce liver toxicity. (**A**) Determination of superoxide dismutase (SOD) activity, (**B**) determination of catalase (CAT) activity, (**C**) determination of glutathione reductase (GSR) activity, and (**D**) determination of malondialdehyde (MDA) activity. The experiments were performed three times or more than three times and data are represented as the mean value ± SEM. A *p*-value less than or equal to 0.05 was considered to be statistically significant, i.e., * *p* ≤ 0.05.

Next, we conducted comparative analysis of antioxidant enzymes (SOD, CAT, and GSR) in the hepatic tissue lysates of animals in all groups. Our results confirmed that a significant decrease in the levels of antioxidant enzymes (SOD, *p* < 0.05; CAT, *p* < 0.05; and GSR, *p* < 0.05) occurred in the group orally administered CCl_4_ (Group B) when compared with the control group (Group A). However, hepatic tissue lysates obtained from the group treated with 50 mg/kg b.w. of TA extract (Group E) exhibited significantly restored antioxidant enzymatic levels when compared with those treated with lower doses of TA extract (30 and 40 mg/kg b.w.; Groups C and D, respectively) and the control group (Group A) (Figure 4A–C). 

The tissue MDA level was verified in the animals of all groups, since it plays a critical role; therefore, the lipid peroxidation process during oxidative stress was induced by CCl_4_. Our results suggest that a significant increase in the MDA level occurred in animals treated with CCl_4_when compared with the control group after the induction of CCl_4-_mediated oxidative stress. However, the hepatic tissue lysates obtained from the TA extract (50 mg/kg b.w.)-treated group significantly decreased the level of MDA, which reflects the reduction in lipid peroxidation when compared with the control group and those treated with lower doses of TA extract (30 and 40 mg/kg b.w.).

### 3.5. TA Extracts Decrease ROS Generation and Offer Protection to Hepatocellular Mitochondria

**Figure 5:**
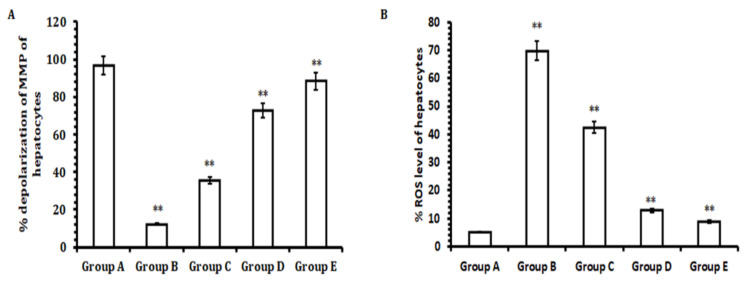
Measurement of MMP and ROS production of liver hepatocytes extracted from the livers of rats initially treated with CCl_4_and the effect of TA extract on MMP and ROS production. (**A**) Measurement of the MMP of hepatocytes extracted from different animal groups initially dosed with CCl_4_, (**B**) ROS production of hepatocytes extracted from different animal groups initially dosed with CCl_4_. The experiments were performed three or more than three times and the data are represented as the mean ± SEM. A *p*-value less than or equal to 0.05 was considered statistically significant, i.e., ** *p* ≤ 0.01.

Under normal physiological conditions, mitochondrial respiration generates some ROS while transferring electrons from adenosine triphosphate to the final acceptor, oxygen [38]. After dosing with CCl_4_orally and reaching the liver hepatocytes, the caused a disruption in the structure, function, and energy production of mitochondria, thereby dissolving lipids and impairing mitochondrial membrane potential. This impairment in the mitochondrial membrane potential led to a disruption in the electron transport chain function, subsequently causing additional ROS to be produced. This excessive ROS production disturbed the antioxidant balance within the hepatocytes and caused necrosis of cells. Our results showed that hepatocytes of the livers of animals initially dosed with CCl_4_hada significantly elevated level of ROS and disruption in the mitochondrial membrane potential when compared with hepatocytes obtained from the livers of animals in the control group. However, the hepatocytes collected from the group treated with 50 mg/kg b.w. of TA extract (Group E) exhibited a remarkable reduction in ROS generation and inhibited depolarization of mitochondrial membrane potential when compared with theCCl_4_-treated group and control group. Conversely, a good reduction in ROS production and the depolarization of mitochondrial membrane potential was also observed in the groups treated with lower doses of TA extract (30 and 40 mg/kg b.w.; Group C and D) when compared with theCCl_4_-treated group (Group B) and control group (Group C) (Figure 5A,B). Together, these results demonstrate that TA extract effectively neutralizes ROS generation, thus aiding in the protection of hepatocytes. It achieves this by reducing the depolarization of the mitochondrial membrane potential.

## 4. Discussion

In the previous study, we reported that TA extract has promising antiproliferative and antioxidant activities. Owing to the presence of high contents of flavonoid and polyphenolic compounds in TA extract [20], the present hypothesis supports the notion that TA extract might have hepatoprotective activity and anti-scavenging effect to restore antioxidant enzymes and neutralize ROS production induced by CCl_4_. Therefore, the current study aimed to evaluate the hepatoprotective activity of TA extract against CCl_4_-mediated hepatotoxicity in Wistar rats. Our results demonstrate that TA extract protects against CCl_4_-mediated hepatotoxicity by restoring serum liver biochemistry, thereby regulating the level of the pro-inflammatory cytokines TNF-α, IL-6, and TGF-β. This was checked by elevating the level of the anti-inflammatory cytokine adiponectin. It attenuates ROS production and maintains the integrity of mitochondrial membrane potential, which ultimately aids in reducing the necrotic population and fibrosis of hepatocytes.

Among the environmental toxicants, CCl_4_is considered one of the prominent toxicants used to study the hepatoprotective effect of various plant-based products for in vivo animal models [39]. CCl_4_, upon administration to the animal body, is converted into the more potent free radical CCl_3_OO^+^ with the help of a group of liver enzyme systems called cytochrome P-450 [40]. This conversion of a toxicant substance into a more toxicant substance by liver enzymes is known as biotransformation [41]. The free radical CCl_3_OO^+^ generated after the biotransformation process in the liver induces lipid peroxidation by interacting covalently with cellular macromolecules (membrane phospholipids) [42]. This interaction causes the disruption of membrane integrity and results in pore formation in the cell membrane, thereby releasing the hepatic enzymes AST, ALP, ALT, and bilirubin from hepatocytes [43]. Thus, CCl_4_administration causes injury to hepatocytes and thereby elevates the levels of liver enzymes in serum [29]. In the current study, we conducted preliminary experiments to evaluate the ideal dose of TA extract and the route of administration in terms of effective hepatoprotective activity with a safe toxicity profile in animals. Our preliminary results suggest that TA extract showed hepatoprotective activity against CCl_4_-mediated liver toxicity in a dose-dependent manner. The optimum activity of TA extract was observed at 50 mg/kg b.w. orally (Group E); the results were significant compared with those obtained with 100 mg/kg b.w. standard silymarin. Additionally, we observed that a dose higher than 60 mg/kg b.w.c aused the animals to show toxicity symptoms, such as lethargy, diarrhea, muscle tremors, loss of appetite, etc., and they died before the completion of the study. The administration of 50 mg/kg b.w. of TA extract (Group E) restored the levels of serum enzymes of the liver to normal by inhibiting the potency of the CCl_3_OO^+^ free radical, which in turn reduced lipid peroxidation, as evidenced by the reduced level of MDA upon the treatment of animals with TA extract compared with those administered CCl_4_ (Group B). The hepatoprotective activity of TA extract was further confirmed by the histopathology results of hepatic tissues. Remarkable hepatocellular damage was observed in animals administered CCl_4_ (Group B) when compared with untreated animals (Group A). Microscopic analysis demonstrated that a significant number of necrotic populations of hepatocytes with large vacuolization, infiltration of inflammatory cells, portal biliary damage, and hepatocyte blooming were observed in animals administeredCCl_4_orally (Group B). However, animals treated with an oral TA extract dose of 50 mg/kg b.w. (Group E) showed significant protection against CCl_4_-mediated hepatotoxicity, which was accompanied by a reduction in the necrosis of hepatocytes, infiltrated cells, less vacuolization, and hepatocyte blooming. 

Another striking feature of hepatic tissue damage caused by CCl_4_is the induction of oxidative stress that affects the levels of antioxidant enzymes, such as SOD, CAT, and GSR [44]. These enzymes together create an anti-scavenging system by catalyzing biochemical reactions to neutralize any free radicals generated under normal physiological conditions into nontoxic compounds to nullify any detrimental effects of toxic compounds, thus helping in the regulation of cell homeostasis [45]. Upon the administration of CCl_4_to animals, the generation of free radicals increased many-fold [46]. This over-production of free radicals caused oxidative stress, which in turn affected the levels of antioxidant enzymes (SOD, CAT, and GSR). However, the animals treated with 50 mg/kg b.w. of TA extract (Group E) exhibited decreased CCl_4_-mediated oxidative stress, specifically by elevating the levels of antioxidant enzymes significantly when compared with the CCl_4_-treated group (Group B). This induction of antioxidant enzymes prevented oxidative stress by neutralizing the CCl_4_-mediated generation of free radicals.

The oxidative stress is induced by CCl_4_-stimulated liver fibrosis in animals dosed with CCl_4_ [47]. As evidenced by the high level of hydroxyproline, a major amino acid was found in collagen tissue by Masson’s trichome staining [48]. Another striking feature of fibrotic tissue was the elevated expression of pro-inflammatory cytokines, which play a crucial role in the stimulation of portal fibroblasts, thereby aiding in the synthesis of more fibrous tissue and associated extracellular matrix substances [49]. Herein, we observed, upon the administration of 50 mg/kg b.w. of TA extract (Group E), significantly decreased fibrosis markers, such as TNF-α, IL-6, and TGF-β, in serum samples when compared with CCL_4_-treated animals (Group B). However, after treating the animals with 50 mg/kg b.w. of TA extract (Group E), we observed a significant increase in the serum level of the anti-inflammatory cytokine adiponectin. Collectively, these results suggest that TA extract has two effects: firstly, it restores the level of anti-inflammatory adiponectin, and secondly, it decreases the level of the pro-inflammatory cytokine proteins TNF-α, IL-6, and TGF-β to control CCl_4_-mediated hepatocellular fibrosis and inflammation.

Besides other factors that cause the necrosis of hepatocytes, CCl_4_administration induces ROS production and mitochondrial membrane potential (MMP) depolarization that lead to hepatic tissue damage and induce the necrosis of hepatocytes [50]. In the current study, our results highlight that hepatocytes of rat livers initially dosed with CCl_4_had significantly elevated levels of ROS and mitochondrial membrane depolarization when compared with hepatocytes obtained from livers from rats in the control group. However, the hepatocytes collected from the animal group treated with TA extract showed a remarkable reduction in ROS generation and inhibited depolarization of mitochondrial membrane potential when compared with the CCl_4_-treated group and control group. Conversely, a significant reduction in ROS generation and the depolarization of mitochondrial membrane potential was observed for the groups treated with lower doses of TA extract (30 and 40 mg/kg b.w.; Groups C and D) when compared with the CCl_4_-treated group (Group B) and control group (Group A). Therefore, in effect, our results demonstrate that TA extract effectively normalizes ROS generation and helps to protect hepatocytes by reducing the depolarization of the mitochondrial membrane potential. Together, these hepatoprotective outcomes may be due to the presence of bioactive compounds in the TA extract. Although the TA extracts exhibited a promising hepatoprotective effect against CCl_4_-induced hepatotoxicity, the limitation of the study was the inability to evaluate the mechanism of action, pharmacokinetics, and bioavailability of its bioactive constituents. 

## 5. Conclusions

Our study demonstrated that TA extract and its major bioactive (polyphenolic and flavonoid) compounds effectively alleviated CCl_4_-induced hepatotoxicity or injury in rat models. More importantly, the hepatoprotective effect of TA extract is mostly attributed to the reduction of ROS, which is associated with oxidative stress, restoration of serum liver biochemistry, inhibition of pro-inflammatory cytokines, and maintenance of mitochondrial membrane potential (Graphical Abstract). Additionally, these results provide a strong platform for the further evaluation of TA extract as a potential agent for treating and preventing liver ailments. 

## Figures and Tables

**Table 1:** Phytochemicals detected from the methanolic extract of TA by LC-MS.

S. No	*M*/*Z*	RT	Compound
1	103.08	6.447481	3-,ethylbutanoic acid;2-methylbutyrate
2	104.04	0.781022	Pyruvate oxime
3	105.12	16.5714	Choline
4	107.04	22.75023	Aromatic aldehyde; D-glycerate
5	108.96	24.27732	Bromoethane
6	119.04	11.48668	Succinic acid
7	126.120003	1.72972046	γ-Coniceine
8	128.04	24.26456	5-amino-4-imidazole carboxylate;1-methyl-4-nitroimidazole
9	130.080002	0.79923889	L-pipecolate
10	131.04	6.248767	Itaconate;(E)-glutaconate
11	133.08	20.91498	(R)-2-hydroxyisocaproate;6-hydroxyhexanoic acid
12	134.04	6.839831	L-aspartate
13	135.12	24.28927	p-cymene
14	136.08	8.631082	2-phenylacetamide
15	137.04	15.86743	Hypoxanthine; threonate
16	139.08	6.450736	4-hydroxyphenylethanol; Styrene-cis-2,3-dihydrodiol
17	140.039993	7.61881435	4-nitrophenol;2-nitrophenol;3-nitrophenol;3-hydroxypicolinic acid
18	142.08	8.676849	Hypoglycin; arecaidine
19	145.08	13.84979	Trans-4-hydroxycyclohexanecarboxylate
20	146.039993	0.78391111	α-ketoglutarate
21	146.16	0.787356	Spermidine
22	147	5.374421	Flupropanate
23	147.12	13.21838	L-lysine
24	149.04	22.59839	D-arabinono-1,4-lactone
25	151.08	4.124735	Tolylacetate
26	160.080002	0.81778333	Indole-3-acetaldehyde
27	161.039993	17.2915444	2-oxoadipate
28	162.119995	6.89406976	L-carnitine
29	163.08	24.28231	Methyl cinnamate; safrole
30	165.12	24.24879	Jasmone
31	189.12	11.11041	Glycyl-leucine
32	167.16	5.766924	Robinobiose
33	175.08	22.49876	N-formimino-L-glutamate
34	177	9.841988	3-Chloro-cis,cis-muconate
35	178.080002	9.75604286	4-hydroxy-4-methylglutamate
36	177.12	10.12313	L-cladinose; metaldehyde
37	181.08	8.720061	D-glucose
38	189.119995	11.1007144	Glycyl-leucine
39	193.080002	5.79493623	Carpacin; myristicin
40	197.160004	8.60402733	Linalyl acetate;alpha-terpinyl acetate
41	199.08	7.810947	L-mimosine
42	205.199997	9.61738333	β-caryophyllene
43	207	26.1537176	Chloroneb
44	207.12	26.1053	1,4-dimethylphenanthrene
45	209.039993	7.79105238	Fraxetin
46	209.16	12.44344	Ammodendrine
47	211.080002	10.4657552	Sedoheptulose
48	219.119995	16.7337904	N-acetylserotonin
49	224.16	8.699876	Tigloidine
50	225.119995	13.7993487	Aspidinol
51	228.119995	20.8984778	Ametryn
52	**229.080002**	**1.01080798**	**Resveratrol**
53	232.080002	6.13259678	N-acetyl-L-2-amino-6-oxopimelate
54	233.160004	10.3468355	Alantolactone
55	241.08	0.891821	(1R,6R)-6-hydroxy-2-succinylcyclohexa-2,4-diene-1-carboxylate
56	245.039993	7.47793282	L-fuculose 1-phosphate
57	245.16	22.48843	Anagyrine
58	248.039993	8.71224279	Pyridoxal phosphate
59	249.12	3.233528	6-hydroxymelatonin
60	254.16	21.66069	Fenapanil
61	**254.279999**	**21.6546583**	**Solenopsin A**
62	256.079987	23.6897444	Apigeninidin
63	257.160004	23.6928028	Chanoclavine-I
64	**265.2**	**24.29171**	**Oxymatrine**
65	266.16	24.28768	Brevicolline
66	270.12	24.60784	Acetochlor
67	276.119995	2.23423865	(5-L-glutamyl)-L-glutamine
68	277.079987	0.93720015	Biotin sulfone
69	282.119995	24.2929528	O6-methyl-2’-deoxyguanosine
70	299.160004	11.7539109	Ostruthin
71	300.119995	12.1321502	2,3,9,10-tetrahydroxyberbine
72	300.96	15.56441	Tolclofos-methyl
73	302.04	15.56511	2-(3,5-dichlorophenylcarbamoyl)-1,2-dimethylcyclopropane-1-carboxylic acid
74	307.079987	9.30033418	Leucocyanidin; gallocatechin
75	307.08	9.293607	Leucocyanidin; gallocatechin; epigallocatechin
76	308.16	5.040135	Tebuconazole
77	311.04	9.821766	Diflubenzuron; edifenphos
78	311.160004	9.16593779	Nafenopin
79	313.08	18.69813	Inosine-5’-carboxylate
80	313.2	18.79439	2,4,6-triphenyl-1-hexene
81	313.32	18.65702	Icosanoic acid
82	314.04	10.93399	Isazofos
83	315.12	18.68262	Dihydropteroate; pyraclonil
84	**315.24**	**18.68325**	**Dronabinol; cannabidiol; cannabichromene**
85	316.08	18.69064	Fenbendazole S-oxide
86	316.2	18.68546	Belladine
87	318	14.04215	Phosmet
88	319.08	11.8284	Lecanoric acid
89	320.16	18.29014	Metconazole
90	321.12	0.803094	Mugineic acid
91	322.079987	2.18447318	β-Citryl-L-glutamate
92	323.04	10.14008	Digallate
93	324.119995	8.44852717	Stylopine
94	324.96	11.66847	Trichloroethanol glucuronide
95	325.2	18.62262	Affinine
96	325.08	13.86467	Sterigmatocystin
97	326.16	20.01459	Monocrotaline
98	327	27.18485	Fenuron
99	327.24	27.23535	12 α-Methylpregna-4,9(11)-diene-3,20-dione
100	328.08	18.00045	Fluazifop
101	328.2	9.572027	Sethoxydim
102	328.32	15.33696	N,N-dimethylsphing-4-enine
103	329.04	11.85282	Fluorodifen
104	329.16	12.03259	1,2-Dehydroreticuline
105	330.12	12.42584	Lithospermoside
106	331.08	13.81768	3,7-Di-O-methylquercetin; cirsiliol; tricin
107	331.2	13.81691	Carnosol
108	336.119995	17.2204175	Trifluralin; befuraline
109	**337.08**	**5.901588**	**Dicumarol**
110	337.200012	5.67431458	Catharanthine; tabersonine
111	337.320007	8.4919533	(13Z,16Z)-docosadienoic acid
112	338.040009	2.47067165	Azafenidin
113	338.88	10.74711	1,2,3,7,8-Pentachlorodibenzofuran
114	339.12	7.357424	Glyceollin I; glyceollin II
115	341.04	24.88731	Diclofop methyl
116	342	24.85467	Bifenox
117	342.12	20.51962	Protodeoxyviolaceinic acid
118	346.079987	12.0925351	Thiamine monophosphate
119	349.200012	11.5365785	Gibberellin A53
120	350.160004	2.21731399	Riddelline
121	353.04	11.55965	Petunidin
122	353.160004	12.090492	Palmatine
123	355.2	26.82419	Vincamine; yohimbine; stemmadenine;
124	355.32	27.48676	22-Oxodocosanoate
125	356.16	27.42916	S-Adenosylmethioninamine
126	357.12	24.57235	Gentiopicrin
127	357.24	24.60749	Arachidonyltrifluoromethane
128	359.04	12.91192	Triflumuron
129	360.12	9.024405	Isopenicillin N
130	360.959991	2.27723168	Chlorthiophos
131	363.12	12.82633	Chelirubine; catalpol
132	364.079987	10.812962	Flufenacet
133	365.04	0.799356	Xanthosine 5’-phosphate
134	365.160004	0.792625	Gibberellin A8
135	367.08	5.559117	Salicin 6-phosphate
136	367.200012	5.68378438	16-Methoxytabersonine; hirsuteine
137	368.040009	9.69718675	Anilofos
137	370.08	14.05884	S-(2-chloroethyl) glutathione
138	371.160004	6.76877846	Bursehernin
139	373.08	10.12554	2-O-caffeoylglucarate
140	373.200012	7.36471102	Biocytin
141	375.119995	23.1907687	Secologanate
142	375.12	13.1768	Portulacaxanthin II; swertiamarin; geniposidic acid; gardoside
143	**377.16**	**8.95533**	**Ailanthone**
144	377.28	19.44733	9-cis-10’-apo-β-carotenal
145	379.079987	0.82208611	7-Methylguanosine 5’-phosphate
146	383.16	3.540126	2-Methoxyestradiol-17β 3-sulfate
147	385.32	22.32738	(8Z,11Z,14Z,17Z,20Z,23Z)-Hexacosahexaenoic acid
148	387	8.48086861	Sulfentrazone
149	387.12	10.09797	1-O-Sinapoyl-β-D-glucose
150	389.160004	10.6243039	Visnadin
151	390.959991	7.31033764	5-Phospho-α-D-ribose 1-diphosphate
152	393.119995	6.13023356	Macarpine
153	395.16	15.13311	Rotenone; deguelin
154	395.399994	17.3745676	Ximenic acid
155	397.32	12.56728	δ-Tocotrienol
156	398.160004	12.6067147	Aureothin
157	399.12	7.147485	S-Adenosyl-4-methylthio-2-oxobutanoate
158	401.28	7.685251	Ophiobolin A
159	403.2	9.7105	Prednisolone acetate; citreoviridin
160	407.16	17.18432	Asperlicin C
161	409.2	9.246711	Abyssinone V
162	410.160004	3.91140291	Linustatin
163	411	11.26515	Imibenconazole
164	411.12	14.16161	Nicosulfuron; spirodiclofen
165	411.24	17.67294	1-Palmitoylglycerol almitoylglycerol 3-phosphate
166	411.36	18.69642	5-Dehydroavenasterol; avenastenone
167	412.320007	23.3697828	Cyclopamine
168	413.04	22.43382	Imazosulfuron; pyraflufen-ethyl
169	413.040009	22.4248369	Pyraflufen-ethyl
170	414.96	17.5999	Flusulfamide
171	415.08	19.1103	(7R)-7-(5-Carboxy-5-oxopentanoyl) aminocephalosporinate
172	415.320007	25.4925863	Yamogenin
173	417.12	11.14687	Daidzin; frangulin A; puerarin
174	418.200012	13.5014327	Casimiroedine
175	423	14.1758789	5-Fluorouridine diphosphate
176	423.119995	22.6974831	Plicatic acid
177	425.279999	17.1062564	α-Phocaecholic acid
178	427.2	12.38413	Abscisic acid glucose ester
179	428.040009	12.3795444	Adenosine 3’,5’-bisphosphate
180	428.16	12.38573	Vicianin
181	429.12	23.93726	ε-Rhodomycinone; ouizalofop-P-tefuryl
182	429.24	23.93274	Nummularine F
183	430.320007	21.7180979	Imperialine
184	437.519989	17.0830779	Hentriacontane
185	438.23999	9.0921256	Lunarine
186	441.12	10.95042	Hallactone B
187	441.36	16.10699	Soyasapogenol C; oleanolic aldehyde
189	443.160004	0.95589167	Mallotochromene
190	447.12	4.350199	Cephamycin C; glycitin
191	453.12	26.06561	Cinchonain 1a
192	453.36	25.52124	Phylloquinol
193	455.160004	14.5180328	ε-Viniferin
194	458.16	20.93682	Amygdalin
195	460.200012	8.64017559	5-Methyltetrahydrofolate
196	461.160004	9.34915583	Paeonolide
197	461.279999	21.0038789	Sophoranone
198	461.399994	9.51068431	Protopanaxadiol
199	**462.959991**	**13.8378296**	**Quercetin 3,3’-bissulfate; quercetin 3,4’-bissulfate**
200	463.079987	14.0931398	Luteolin 7-O-glucuronide
201	463.200012	16.7546761	Plicamine
202	467.160004	22.6358392	Agnuside
203	467.279999	20.3436707	Cephaeline
204	471.36	21.25501	Gypsogenin
205	473.399994	22.5188859	α-Tocopherol acetate
206	474.12	16.33285	CMP-N-trimethyl-2-aminoethylphosphonate
207	485.28	25.71086	Stigmatellin
208	486.96	5.876778	5-Fluorodeoxyuridine triphosphate
209	487.200012	23.1899191	Rutaevin; haplodimerine; nafenopin glucuronide
210	489.36	24.9554	Asiatic acid
211	489.12	9.603542	CDP-choline
212	492	10.758352	dATP
213	495.119995	23.7432148	5’-Methoxyhydnocarpin-D
214	497.279999	23.6242253	Absinthin
215	**517.2**	**22.54293**	**Rottlerin**
216	517.320007	23.5029426	Cucurbitacin D
217	525.47998	22.7749935	Retinyl palmitate
218	541.200012	21.6297253	Oleuropein
219	553.44	24.15608	Zeinoxanthin; β-cryptoxanthin
220	593.400024	24.1761293	Santiaguine
221	625.200012	13.1988915	Verbascoside
222	629.280029	23.81974	Resiniferatoxin

**Table 2:** Major compounds of *Tamarix articulata* exhibiting antitumor activity.

S. No	Major Compounds	Functions	References
**1.**	Solenopsin	Exhibits antiproliferative activity and inhibits PI3K/Akt-driven angiogenesis	[30]
**2.**	Ailanthone	Promotes apoptosis and autophagy by increasing the expression of miR-195 in leukemia cells	[31]
**3.**	Dicumarol	Exhibits promising antiproliferative effects and induces ROS-mediated mitochondria-dependent apoptosis in breast cancer cells	[32]
**4.**	Dronabinol	Promotes antiproliferative activity and induces apoptosis by cannabinoid receptor (CB) ½ in leukemia cells	[33]
**5.**	Oxymatrine	Induces apoptotic cell death and arrests the G0/G1 cell cycle phase by blocking EGFR/PI3K/Akt/mTOR signaling in glioma cells	[34]
**6.**	Quercetin	Immuno-modulatory effect, anticancer, anti-inflammatory, and antiviral activities	[35]
**7.**	Resveratrol	Exhibits antioxidant, anti-inflammatory, and antiproliferative activities, interferes in many signaling pathways, and activates apoptosis in cancer cell models	[36]
**8.**	Rottlerin	Antihypertensive, antiallergic, and antifertility activities. Promotes antiproliferative activity by downregulating NF-kB and cyclin D1 expression	[37]

**Table 3:** Dose optimization of TA extract. Forty-two Wistar male rats were divided into seven groups containing six animals each. Group A was designated as normal without any chemical or extract treatment. Group B was designated as the CCl_4_-treated group and the animals were administered 40% CCl_4_mixed in olive oil orally for 3 alternate days a week for 8 weeks. Groups C, D, E, and F were administered 40% CCl_4_mixed in olive oil and 30, 40, 50, and 60 mg/kg b.w. TA extract, respectively, and all treatments were administered for 3 alternate days a week for 8 weeks. Group G was designated as 40% CCl_4_mixed in olive oil and standard silymarin 100 mg/kg orally for 3 alternate days a week for 8 weeks.

Groups	Treatments/Dosages	Schedules (200 µL)
A	Control (without CCl_4_)	Vehicle treatment (after 8 weeks animals were sacrificed)
B	CCl_4_	CCl_4_ (diluted 40%:60% in olive oil)/100 g rat, orally, thrice weekly, for 8 weeks; after 8 weeks, the animals were sacrificed
C	CCl_4_ + TA extract (30 mg/kg b.w.)	CCl_4_ (diluted 40%:60% in olive oil)/100 g rat, orally, thrice weekly. TA extract (30 mg/kg p.o.; 1 mL/100 g body weight) thrice weekly for 8 weeks; after 8 weeks, the animals were sacrificed
D	CCl_4_ + TA extract (40 mg/kg b.w.)	CCl_4_ (diluted 40%:60% in olive oil)/100 g rat, orally, thrice weekly. TA extract (40 mg/kg p.o.; 1 mL/100 g body weight) thrice weekly for 8 weeks; after 8 weeks the animals were sacrificed
E	CCl_4_ + TA extract (50 mg/kg b.w.)	CCl_4_ (diluted 40%:60% in olive oil)/100 g rat, orally, thrice weekly. TA extract (50 mg/kg p.o.; 1 mL/100 g body weight) thrice weekly for 8 weeks; after 8 weeks, the animals were sacrificed
F	CCl_4_ + TA extract (60 mg/kg b.w.)	CCl_4_ (diluted 40%:60% in olive oil)/100 g rat, orally, thrice weekly. TA extract (60 mg/kg p.o.; 1 mL/100 g body weight) thrice weekly for 8 weeks; after 8 weeks, the animals were sacrificed
G	CCl_4_ + Silymarin (100 mg/kg b.w.)	CCl_4_ (diluted 40%:60% in olive oil)/100 g rat, orally, thrice weekly. Silymarin (100 mg/kg p.o.; 1 mL/100 g body weight) thrice weekly for 8 weeks; after 8 weeks, the animals were sacrificed

**Table 4:** Analysis of the antioxidant enzymes (SOD, CAT, GSR, and MDA) of liver tissues initially treated with CCl_4_to induce liver toxicity and treatment with varying doses of TA extract (30, 40, 50, and 60 mg/kg b.w.) along with standard silymarin (100 mg/kg b.w.). The data represent the mean value ±SEM of three independent experiments. * *p*< 0.05, *** *p*< 0.001, and **** *p*< 0.0001.

Groups	Treatment/	SOD (U/mg Protein)	CAT (U/mg Protein)	GSR (nmol/mg Protein)	TBARS (nmol MDA/mg Protein)
	Dosage				
A	Control (without CCl_4_)	29.64 ± 0.16	146.01 ± 2.87	22.45 ± 0.35	0.457 ± 0.01
B	CCl_4_	10.67 ± 0.14 ****	79.76 ± 1.02 ****	7.87 ± 0.16 ****	1.18 ± 0.09 ****
C	CCl_4_ + TA extract (30 mg/kg b.w.)	13.59 ± 0.56 ****	108.93 ± 1.91 ****	12.09 ± 0.15 ****	0.98 ± 0.03 ****
D	CCl_4_ + TA extract (40 mg/kg b.w.)	22.94 ± 0.53 ****	119.98 ± 2.08 ****	16.34 ± 0.42 ****	0.73 ± 0.04 ****
E	CCl_4_ + TA extract (50 mg/kg b.w.)	27.78 ± 0.53 *	145.97 ± 1.08 *	21.91 ± 0.09 *	0.49 ± 0.03 *
F	CCl_4_ + TA extract (60 mg/kg b.w.)	Animals died before the completion of the study	Animals died before the completion of the study	Animals died before the completion of the study	Animals died before the completion of the study
G	CCl_4_ + Silymarin (100 mg/kg b.w.)	28.63 ± 0.43 ***	149.02 ± 3.78 ****	22.40 ± 0.36 ****	0.39 ± 0.9 ****

**Table 5:** Analysis of the serum biochemical parameters in the liver function test after CCl_4_-induced liver toxicity and treatment with varying doses of TA extract (30, 40, 50, and 60 mg/kg b.w.) along with standard silymarin (100 mg/kg b.w.). The data represent the mean value ±SEM of three independent experiments. * *p*< 0.05, and **** *p*< 0.0001.

Groups	Treatment	AST (UL^−1^)	ALT (UL^−1^)	ALP (UL^−1^)	Bil (µmol L^−1^)
A	Control (without CCl_4_)	32.31 ± 0.98	66.01 ± 2.87	498.98 ± 20.45	1.23 ± 0.11
B	CCl_4_	2945 ± 82.54 ****	388.66 ± 12.02 ****	865 ± 17.76 ****	3.98± 0.23 ****
C	CCl_4_ + TA extract (30 mg/kg b.w.)	576.12 ± 33.56 ****	288.93 ± 16.91 ****	509.19 ± 34.85 ****	1.58 ± 0.19 ****
D	CCl_4_ + TA extract (40 mg/kg b.w.)	271.83 ± 19.53 ****	159.98 ± 07.58 ****	456.34 ± 16.42 ****	1. 43 ± 0.04 ****
E	CCl_4_ + TA extract (50 mg/kg b.w.)	172.78 ± 12.73 *	65.97 ± 1.08 *	391.94 ± 23.02 *	1. 19 ± 0.03 *
F	CCl_4_ + TA extract (60 mg/kg b.w.)	Animals died before the completion of the study	Animals died before the completion of the study	Animals died before the completion of the study	Animals died before the completion of the study
G	CCl_4_ + Silymarin (100 mg/kg b.w.)	170.63 ± 10.03 ****	69.02 ± 3.78 ****	403.40 ± 13.56 ****	1. 29 ± 0.43 ****

**Table 6:** Histopathological changes and grading of micro-sections of the liver.

Groups	Hepatocyte Ballooning	Hepatocyte Necrosis	Fatty Changes (Lipidosis)	Inflammatory Cells Infiltration	Portal Fibrosis
A	0	+	0	+	0
B	+++	+++++	+++++	++++	+++
C	+	+++	+++	++	++
D	+	++	++	+	+
E	+	+	+	+	+

Histological changes in micro-sections of liver were scored for hepatic injury through light microscopy, with 0 = visibly no cellular damage, + = focal hepatocyte damage (less than 10–20%), ++ = focal hepatocyte damage (20–40%), +++ = extensive focal hepatic damage, ++++ = extensive focal hepatic damage with lesions, and +++++ = global hepatocyte necrosis.

## Data Availability

Data exists within the article.

## Abbreviations

TA, *Tamarix articulata*; CCl_4_, carbon tetrachloride; SOD, superoxide dismutase; CAT, catalase; GSR, glutathione reductase; TBARS, thiobarbituric reactive substances; TNF-α, tumor-necrosis factor-alpha; TGF-β, tumor-growth factor-beta; IL-6, interleukin-6; ROS, reactive oxygen species; CCl_3_^+^, trichloromethyl radical; CCl_3_OO^+^, trichloromethylperoxy; LFT, liver function test; MMP, mitochondrial membrane potential; LC–MS, liquid chromatography–mass spectrometry; ESI-MS, electrospray ionization mass spectra; KAUST, King Abdullah University of Science and Technology; ALP, alkaline phosphatase; ALT, alanine transaminase; AS, aspartate aminotransferase; ELISA, enzyme-linked immunosorbent assay; PBS, phosphate-buffered saline; MDA, malondialdehyde; DCFH-DA, 2′-7′dichlorofluorescin diacetate.
